# In Situ High-Pressure X-ray Diffraction and Raman Spectroscopy Study of Ti_3_C_2_T_*x*_ MXene

**DOI:** 10.1186/s11671-018-2746-4

**Published:** 2018-10-29

**Authors:** Luxi Zhang, Weitao Su, Yanwei Huang, He Li, Li Fu, Kaixin Song, Xiwei Huang, Jinhong Yu, Cheng-Te Lin

**Affiliations:** 1 College of Materials and Environmental Engineering, Hangzhou Dianzi University, Hangzhou, 310018 China; 2 Center for High Pressure Science and Technology Advanced Research, Shanghai, 201203 China; 3 College of electronics and information, Hangzhou Dianzi University, Hangzhou, 310018 China; 4 Key Laboratory of Marine Materials and Related Technologies, Zhejiang Key Laboratory of Marine Materials and Protective Technologies, Ningbo Institute of Materials Technology and Engineering, Chinese Academy of Sciences, Ningbo, 315201 China

**Keywords:** Ti_3_C_2_T_*x*_ MXene, High-pressure XRD, High-pressure Raman, Grüneisen parameter

## Abstract

The lattice stability and phonon response of Ti_3_C_2_T_*x*_ MXene at high pressure are important for understanding its mechanical and thermal properties fully. Here, we use in situ high hydrostatic pressure X-ray diffraction (XRD) and Raman spectroscopy to study the lattice deformation and phonon behavior of Ti_3_C_2_T_*x*_ MXene. XRD spectra indicate that no phase transformation occurs up to the pressure of 26.7 GPa. The elastic constant along *a* lattice parameter was calculated to be 378 GPa. In the Raman spectra obtained at high-pressure, the out-of-plane phonon modes (*A*_*1g*_ at ~ 210, ~ 504, and ~ 711 cm^−1^) exhibit monotonic blueshifts with increasing pressure. The Grüneisen parameters of these three modes were calculated to be 1.08, 1.16, and 0.29, respectively. These results enrich the basic property data of Ti_3_C_2_T_*x*_ MXene and would benefit the further understanding of this novel material.

## Background

After the intensive studies of graphene [1] and transition metal dichalcogenides(TMDs) [2–5] for a decade, two-dimensional (2D) metal carbides (MXenes) have been drawing much attention recently owing to their extraordinary electrical properties [6, 7]. The Ti_3_C_2_ MXene is a layered material with Van der Waals stacked structure, in which each layer contains two carbon atom planes sandwiched among three Ti atom planes. In the energy band structure of Ti_3_C_2_ MXene, the conduction band touches the valence band at the Γ point, which indicates that Ti_3_C_2_ MXene is a half-metallic material [8]. More interestingly, the band structure of Ti_3_C_2_ Mxene can be opened up slightly by the surface functional groups (which are annotated as “T” in the formula) [8], such as –F, –O, and –OH [9]. These functional groups are generated in the solution etching of M_*n*_AlC_*n* + 1_ phase [9], which then forms a Ti_3_C_2_T_*x*_ MXene with tunable electrical properties. The electrical conductivity of Ti_3_C_2_T_*x*_ MXene was measured to be 4.2 × 10^−4^S/m [10], which is superior to most TMDs. To date, Ti_3_C_2_T_*x*_ MXenes have shown potential applications in advanced supercapacitors [11], Li-batteries [12], electromagnetic shielding [10], antibacterial [13]**,** and light emission [14].

In addition to high electrical conductivity, the elastic property of Ti_3_C_2_T_*x*_ MXene also attracts much attention. Theoretical calculations predicted that this ultrathin carbide has a Young’s modulus of as high as ~ 500 GPa [15–17]. Lipatov et al. recently used nanoindentation to determine that the Young’s modulus of monolayer Ti_3_C_2_T_*x*_ MXene was 330 Gpa [18]. These values are much higher than those of MoS_2_ [18] and are comparable to those of monolayer graphene [19]. Recently, Ghidiu et al. measured the high-pressure X-ray diffraction (XRD) spectra of Ti_3_C_2_T_*x*_ MXene up to 3 GPa and observed no phase transformation [20]. However, as the pressure loaded in ref. [20] was too low, the phase stability and lattice deformation of Ti_3_C_2_T_*x*_ at higher pressure are still unknown.

Raman spectroscopy acts as a useful non-destructive tool to investigate the crystal structure and phonon vibration of 2D materials such as graphene [21] and TMDs [2]. The composition of Ti_2_CT_*x*_ [22] and phase stability of Ti_3_C_2_T_*x*_ Mxene at different annealing conditions [10] can be probed by using confocal Raman measurements. Recently, the phonon dispersion of Ti_3_C_2_T_*x*_ MXene was theoretically calculated by Hu et al. [23, 24], thus enabling a further understanding of the Raman spectra of this material. However, the high-pressure Raman spectroscopy of Ti_3_C_2_T_*x*_ is still lacking. Moreover, the phonon response of Ti_3_C_2_T_*x*_ as a function of pressure is unknown.

In this paper, we prepared Ti_3_C_2_T_*x*_ thin flakes and measured their pressure-dependent XRD and Raman spectra up to 26.7 GPa. The elastic constants of Ti_3_C_2_T_*x*_ were calculated from XRD diffraction peak shifts by the Murnaghan equation. The positive Grüneisen parameters of out-of-plane phonons were obtained from their pressure-dependent Raman shift and lattice parameter deformation ratio. The obtained results would benefit the further understanding of the mechanical and phonon-vibrational behavior of Ti_3_C_2_T_*x*_ MXene.

## Results and Discussions

**Fig. 1:**
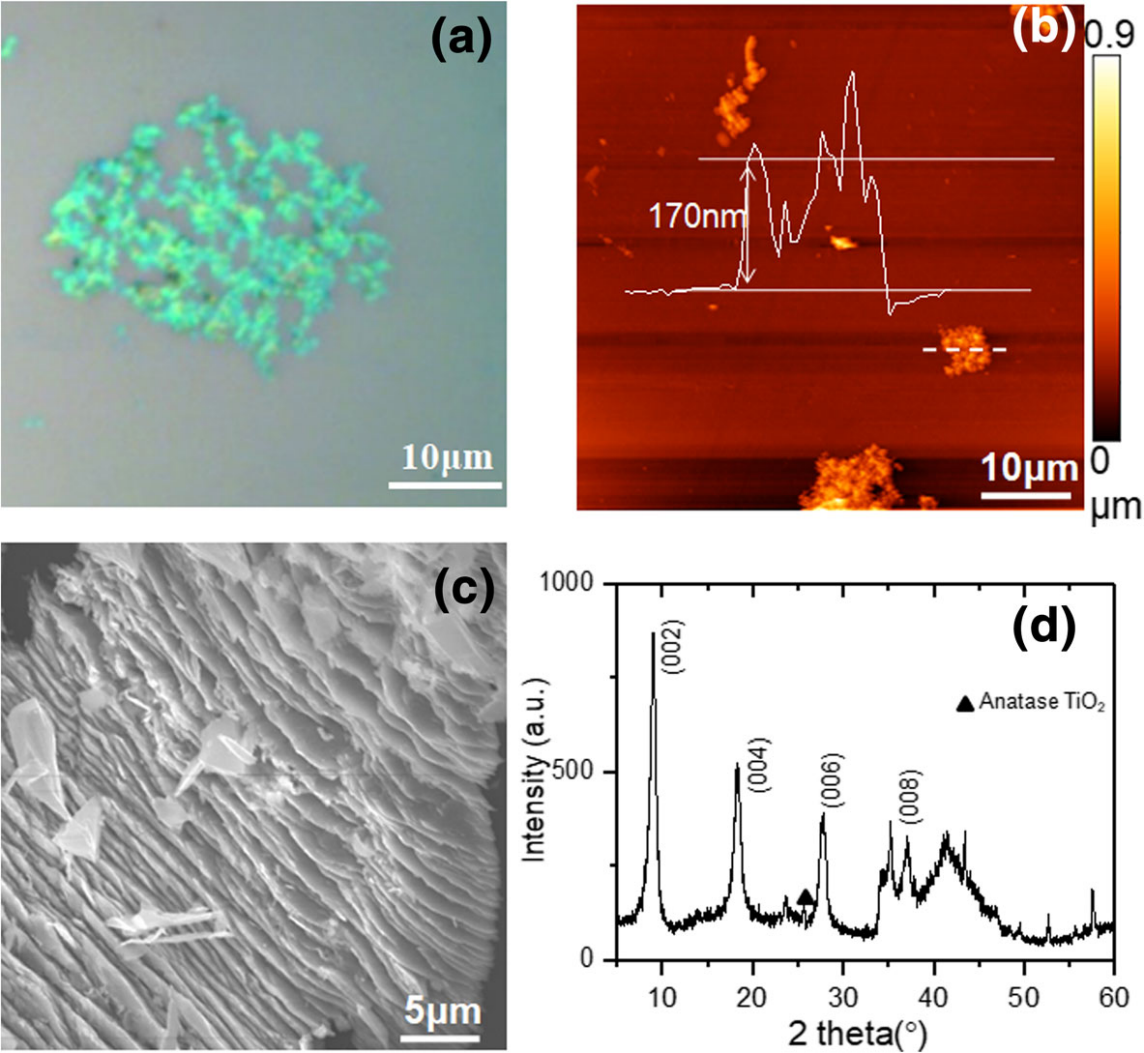
**a** Optical image of ultrasonically exfoliated Ti_3_C_2_T_*x*_ flakes; **b** AFM topographic image of ultrasonically exfoliated Ti_3_C_2_T_*x*_ flakes, and a line profile across the marked dashed line is shown as an inset, indicating the Ti_3_C_2_T_*x*_ flake thickness of 170 nm; **c** SEM image of ultrasonically exfoliated Ti_3_C_2_T_*x*_ flakes; **d** XRD spectra of Ti_3_C_2_T_*x*_ raw powder

Before conducting high-pressure measurements, we first investigated the basic material properties of the exfoliated Ti_3_C_2_T_*x*_ Mxene flakes. An optical image of the exfoliated Ti_3_C_2_T_*x*_ flakes deposited on Si/SiO_2_ (300 nm) substrate is shown in Fig. 1a. Light green contrast can be observed for the exfoliated flakes. As reported by Miranda et al., the optical contrast of Ti_3_C_2_T_*x*_ flakes depends on the flake thickness strongly, where thicker flakes always show higher contrast, while thin flakes exhibit low contrast [25]. The light green contrast of most of the flakes in Fig. 1b indicates their thin thickness. An atomic force microscope (AFM) topographic image of exfoliated Ti_3_C_2_T_*x*_ flakes is shown in Fig. 1b. The flakes in the mapping area show surfaces with high roughness, which is typical for Ti_3_C_2_T_*x*_ flakes [26]. The thickness of a typical thin flake can be determined from its line profile (inset of Fig. 1b) across the marked position in Fig. 1b to be 170 nm. A scanning electron microscope (SEM) image of an exfoliated flake is shown in Fig. 1c. The laminated structure of Ti_3_C_2_T_*x*_ can be seen clearly, indicating the successful preparation of Ti_3_C_2_T_*x*_ layered samples [10].

We further measured the XRD spectra of raw Ti_3_C_2_T_*x*_ powder, as shown in Fig. 1d. This XRD pattern is an analogy to previous reports [10]; thus, the prominent peaks at 8.95°, 18.28°, and 27.7° can be assigned to the diffractions of (002), (004), and (006) planes. Compared to the prominent peaks, the intensity of diffraction peaks of minor phases (anatase TiO_2_(101) at 25.3°, JCPDS Card No. 71-1116) is relatively weak, indicating the high purity of Ti_3_C_2_T_*x*_ phase in the obtained powder. The (002) peak emerges at an angle slightly lower than that reported by Han et al. (9.21°) [10]. The calculated *c* lattice parameter, 19.66 Å, is larger than the reported value (19.2 Å) [10]. It should be noted that since the interlayer space may be tuned by different densities of chemical groups and ions, such as –F, –OH, and Li^+^, the *c* lattice parameter varies substantially from 19.2 Å to 58.8 Å in different studies [10, 20, 26, 27]. The *c* lattice parameter for our sample is very close to the low value that was measured for Ti_3_C_2_T_*x*_ powder simply using HF as etchant [10].

**Fig. 2:**
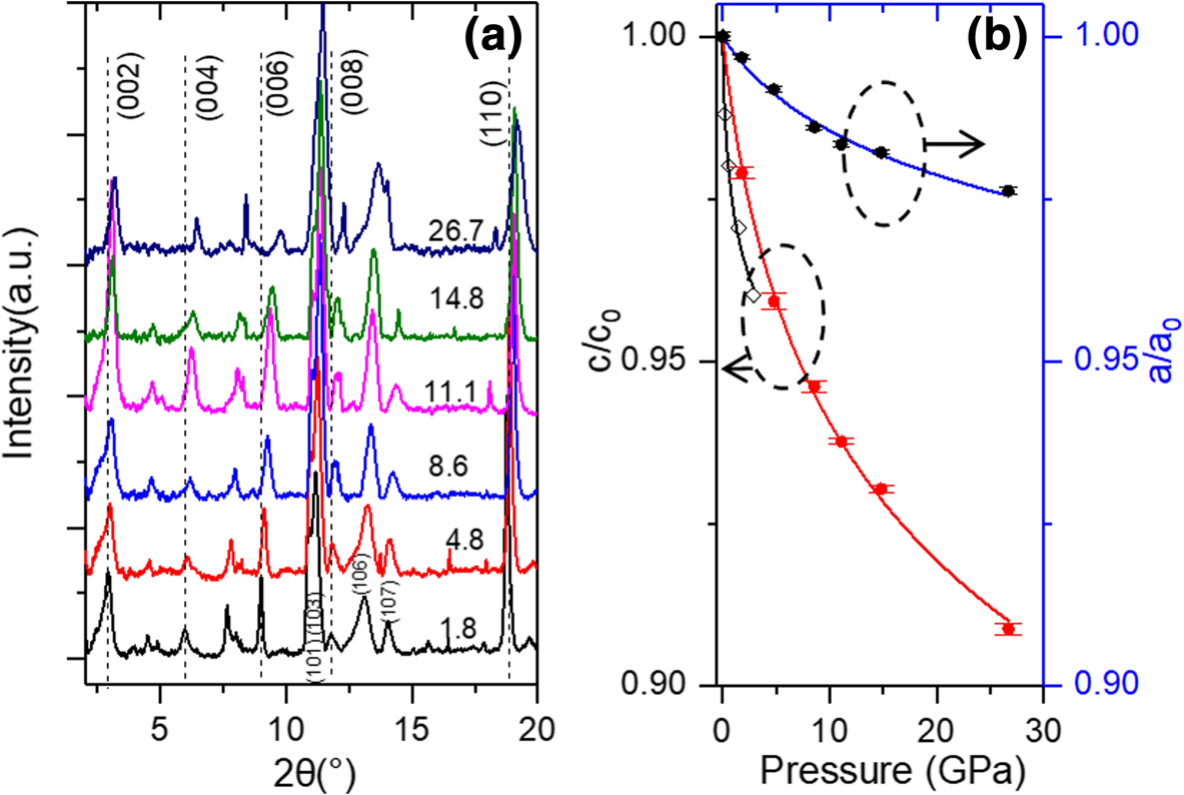
**a** XRD spectra of Ti_3_C_2_T_*x*_ at different loaded pressures. Note that the unit is GPa for the pressure annotated on each spectrum. Peaks are assigned according to ref. [26]; **b** experimental (dots) and calculated compressive ratio (solid line) along *c* and *a* directions. The solid lines are the fitted results using the equation $r(P)/{r}_0={\left[\left({\delta}_0/{\delta}^{\prime}\right)P+1\right]}^{\delta^{\prime }}$

The XRD spectra of Ti_3_C_2_T_*x*_ flakes measured at different pressures until 26.7 GPa are shown in Fig. 2a. It can be seen that the spectra measured at different pressures are similar to each other, while no new diffraction peak can be found. This finding indicates no phase transformation occurs with the pressure up to 26.7 GPa. In Fig. 2a, all the diffraction peaks shift to large angles with increasing pressure, indicating the shrinkage of the Ti_3_C_2_T_*x*_ lattice. Such pseudo-negative compressibility has also been observed for Ti_3_C_2_T_*x*_ [20] and other low dimensional materials with a layered structure, such as graphite [28], graphene oxide [29, 30], MoS_2_ [31], clay [32], and titanates [33]. The (002) peak shifts from 2.883° to 3.162° as the pressure increased from 1.8 GPa to 26.7 GPa. The deformation ratio of lattice parameter *c*, *c*/*c*_*0*_, as a function of pressure, can be calculated from the shift of (002) peak. Moreover, the deformation ratio of *a*, *a*/*a*_*0*_, can be calculated from the shift of (110) peak. As shown in Fig. 2b, the lattice parameters *c* and *a* are deformed by 9.1% and 2.4%, respectively, at a pressure of 26.7 GPa. In the low-pressure region at ~ 3 GPa, the compressive ratio of lattice parameter *c* is 3%. In the previous high-pressure XRD measurement of Ti_3_C_2_T_*x*_ flakes, a slightly larger *c* compressive ratio of 4% for dry Ti_3_C_2_T_*x*_ flakes was reported by Ghidiu et al. [11](Fig. 2b). This difference could be induced by a larger lattice parameter *c*(25.1 Å) of the sample used by Ghidiu et al. [11] with respect to that of ours(19.66 Å).

To obtain the elastic constants, the *c* and *a* compressive ratios in Fig. 2b are further fitted by using the Murnaghan equation [34].

1 $$ r(P)/{r}_0={\left[\left({\beta}^{\prime }/{\beta}_0\right)P+1\right]}^{-1/{\beta}^{\prime }} $$

where *r* represents the lattice constants along *c* and *a* axes, ${\beta}_0^{-1}=-{\left(\frac{dlnr}{lnP}\right)}_{P=0}$ is the linear compressibility, and *β*^*′*^ is the pressure derivate of *β*.

The fitted *r*/*r*_*0*_ curves of *a* and *c* are plotted as solid lines in Fig. 2b. It can be seen that the experimental results fit the equation expectation very well. The best fitting generates *β*_*0*_ and *β*^*′*^ for *c* as 67.7 GPa and 25.5, respectively. While for lattice parameter *a*, *β*_*0*_ and *β*^*′*^ are calculated to be 387.4 GPa and 72.1, respectively (Table 1). For ultrathin 2D materials such as graphene, Young’s modulus (1TPa) is very close to the *β*_*0*_ of thick graphite [19, 28]. Therefore, *β*_*0*_ can be used as a substitution to evaluate the elastic constant of Ti_3_C_2_T_*x*_. Young’s modulus of Ti_3_C_2_T_*x*_ was recently measured by Lipatov et al. to be 330 GPa [18], which is consistent with the *β*_*0*_ in our study. Our measured values are also comparable to the elastic constants of Ti_3_C_2_ that were calculated in other studies [15, 17] (Table 1). The *β*_*0*_ at *c* axis is larger than that of graphite (*β*_*0*_ *=* 35.7 GPa), while the *β*_*0*_ at *a* axis is smaller than that of graphite (*β*_*0*_ *=* 1250 GPa) [28]. The *β*_*0*_ of Ti_3_C_2_T_*x*_ is higher than the bulk modulus of MoS_2_ (270 Pa) [35] and is also comparable to that of graphene oxide (210 GPa) [36], indicating a high elastic constant of Ti_3_C_2_T_*x*_ Mxene among 2D materials.

**Table 1:** The first and second order compression coefficients of Ti_3_C_2_T_*x*_, which are calculated by fitting Fig. 2b through Eq. 1

Mode	*β*_*0*_ (cm^−1^)	*β’*	
*c* axis	67.7	25.5	This study
*a* axis	387.4	72.1	This study
	502 (Young’s modulus)		Borysiuk et al. [16]
	473 (C_11_)		Ning et al. [17]
	491 (C_11_)		Bai et al. [15]
	447 (Young’s modulus)		Ning et al. [17]
	330 (Young’s modulus)		Lipatov et al. [18]

**Fig. 3:**
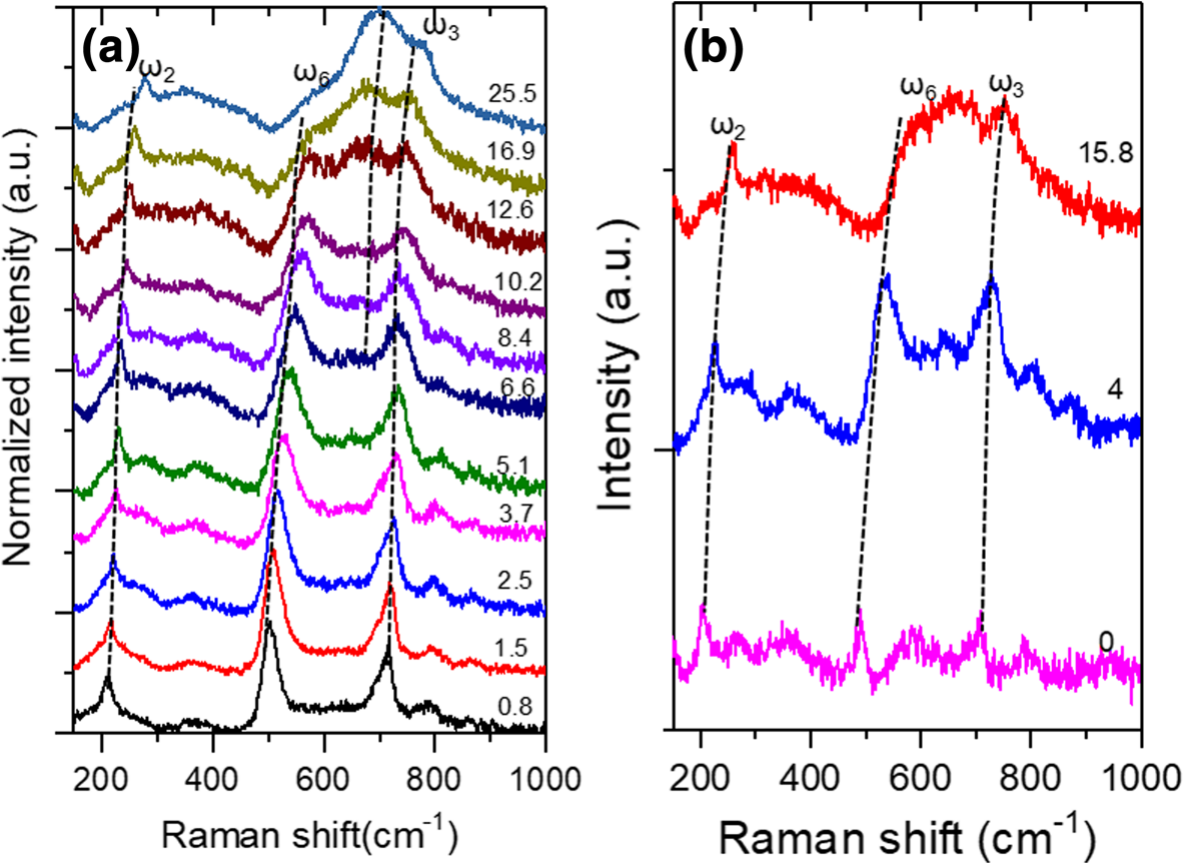
**a** Raman spectra of Ti_3_C_2_T_*x*_ flakes at different compression pressures; **b** Raman spectra obtained at different decompression pressures. Note that the unit of pressures in **a** and **b** is GPa

High-pressure Raman spectra of Ti_3_C_2_T_*x*_ samples were measured at different compressive pressures up to 25.5 GPa, as shown in Fig. 3a. The Raman spectra obtained at different decompressive pressures are shown in Fig. 3b. At low compressive pressures, Ti_3_C_2_T_*x*_ Mxene exhibits three major Raman bands at ~ 210, ~ 500, and 700 cm^−1^. It should be noted that the Raman spectra of Ti_3_C_2_T_*x*_ MXene vary significantly in different kinds of literature. Hu et al. [23] reported strong Raman peaks at ~ 200 cm^−1^ and 720 cm^−1^, while other bands at 400 cm^−1^ were quite broad. However, Han et al. [10] and Zhu et al. [37] observed a sharp peak at ~ 200 cm^−1^, but other bands were all broad. Xue et al. [14] only observed broad peaks from 100 to 700 cm^−1^. The Raman spectra in Fig. 3 are different from those in ref. [10, 14, 23, 37]. This difference could be induced by different types and concentrations of chemical groups on Ti_3_C_2_T_*x*_ MXene. Further interpretation of these different Raman bands needs to recall the phonon dispersion of Ti_3_C_2_T_*x*_ that was theoretically calculated by Hu et al. [23, 24]. The space group of Ti_3_C_2_T_*x*_ was P6_3_/mmc [23]. The number of atoms (*N*) in a primitive cell of Ti_3_C_2_T_*x*_ was calculated to be 7, 7, and 9 for T = −O, −F, and −OH, respectively, given *x* = 2. At *Γ* point of the first Brillouin zone, the following optical phonons are predicted to be existing for different Ti_3_C_2_T_*x*_ MXenes: *Γ*_optical_ (Ti_3_C_2_O_2_) = 6*E*_*g*_ + 3*A*_*1g*_, *Γ*_optical_ (Ti_3_C_2_F_2_, Ti_3_C_2_ (OH)_2_) = 8*E*_*g*_ + 4*A*_*1g*_ [23]. The atom vibrations of different Raman active modes of Ti_3_C_2_F_2_ and Ti_3_C_2_(OH)_2_ are illustrated schematically in Table 2. Their frequencies were theoretically calculated by Hu et al. [23] and are listed in Table 2. For Ti_3_C_2_(OH)_2_, there are four out-of-plane modes (*A*_*1g*_: 218, 514, 684, and 3734 cm^−1^) and four in-plane modes (*E*_*g*_: 138, 278, 437, and 622 cm^−1^). For Ti_3_C_2_F_2_, there are three *A*_*1g*_ modes (190, 465, and 694 cm^− 1^) and three *E*_*g*_ modes (128, 231, and 612 cm^− 1^) [23].

**Table 2:** Assignment of typical Raman peaks in this work and reference. The theoretically calculated phonon energies of Ti_3_C_2_(OH)_2_ and Ti_3_C_2_F_2_ are listed for comparison. The vibration directions of atoms in a unit cell are also shown schematically. The unit of Raman shift is cm^−1^

	ω_1_ (*E*_*g*_)	ω_2_ (*A*_*1g*_)	ω_3_ (*A*_*1g*_)	ω_4_ (*E*_*g*_)	ω_5_ (*E*_*g*_)	ω_6_ (*A*_*1g*_)	ω_7_ (*E*_*g*_)
This work		205.6	702.5			490.2	
Ref [10, 23]		210	730	630			380
Ti_3_C_2_(OH)_2_ [23]	138	218	684	622	278	514	437
Ti_3_C_2_F_2_ [23]	128	190	694	612	231	465	

However, it can be seen the calculated phonon frequencies of pure Ti_3_C_2_F_2_ or Ti_3_C_2_(OH)_2_ cannot fit the experimental Raman spectra of Ti_3_C_2_T_*x*_ in Fig. 3. Because the surface of Ti_3_C_2_T_*x*_ is usually attached by a different type of chemical groups, a full interpretation of the experimental Raman spectra in Fig. 3 needs to consider the hybridized vibration modes of −F and −OH [23]. In a previous research [23], the Raman bands at ~ 200, ~ 500, and ~ 700 cm^−1^ were assigned to ω_2_, ω_6_, and ω_3_, respectively. Following this instruction, the prominent Raman bands at 205.6, 490.2, and 702.5 cm^−1^ of Fig. 3a, b can be assigned to ω_2_, ω_6_, and ω_3_ modes, respectively. Interestingly, these modes are all out-of-plane modes. Other Raman modes are difficult to be isolated from their neighboring modes due to their low intensities. To obtain robust data by eliminating the uncertainties, only these three modes are considered in the following calculations and analyses.

In Fig. 3a, b, it can also be seen that the relative intensities of these in-plane modes increase with the increasing compressive pressures (Fig. 3a). When the compressive pressure is ≥ 12.6 GPa, a new peak at ~ 600 cm^−1^ (ω_4_) emerges and becomes the prominent peak. In the decompression process, the intensities of this ω_4_ mode decrease significantly. The Raman spectra obtained at 0 GPa of decompression pressure contain almost all the in-plane and out-of-plane phonon modes. Such emergence of in-plane modes at high compressive pressure might be related with flake fracture or orientation rotation-induced polarization. Research about this effect is still ongoing and will be reported in the future.

**Fig. 4:**
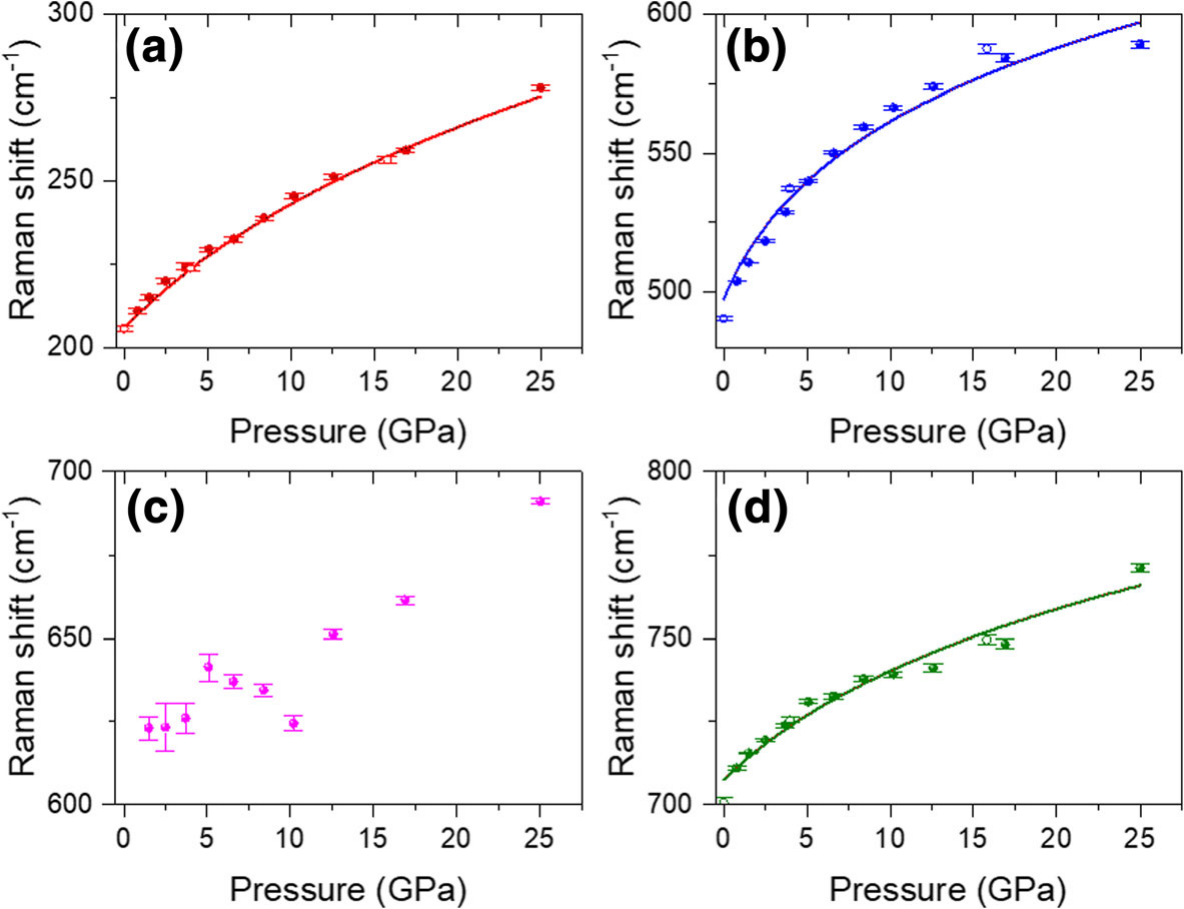
Raman shifts of different phonon modes as a function of different compressive pressures (solid sphere) and decompressive pressures (opened circles): **a** 210 cm^−1^, **b** 504 cm^−1^, **c** 620 cm^−1^, and **d** 711 cm^−1^. Solid lines are the fitting results using the equation $\omega (P)/{\omega}_0={\left[\left({\delta}_0/{\delta}^{\prime}\right)P+1\right]}^{\delta^{\prime }}$

With increasing pressure from 0.8 GPa to 25.6 GPa, ω_2_, ω_6_, and ω_3_ all show monotonic increasing blueshifts (Fig. 4a–d), which are similar to the pressure-dependent blueshifts of graphite [28] and MoS_2_ [31]. At 25.6 GPa, these three modes’ blueshifts are 66.7, 85.1, and 60 cm^−1^, respectively. Such pressure-dependent blueshifts are much larger than those of MoS_2_ [31]. To quantify the Raman shift vs pressure, the Raman shift plots in Fig. 4a, b, d were fitted using the following equation [28]:

2 $$ \omega (P)/{\omega}_0={\left[\left({\delta}_0/{\delta}^{\prime}\right)P+1\right]}^{\delta^{\prime }} $$

where *δ*_0_ and *δ*^′^ are the logarithmic pressure derivative(*dlnω*/*dP*)_*P* = 0_ and the pressure derivative of *dlnω*/*dP*, respectively. The fitted results are plotted as solid lines in Fig. 4a, b, d. Due to the high uncertainties at the low-pressure region, the Raman mode at 620 cm^−1^ was not fitted. It can be seen in Fig. 4a, b, d that the fitted curves are consistent with the experimental results, indicating the high accuracy of the fitting process. The obtained parameters of *δ*_0_ and *δ*^′^ are listed in Table 3.

**Table 3:** Calculated pressure-dependent parameters of Ti_3_C_2_T_*x*_. $\overline{\upgamma}$ refers to the averaged Grüneisen parameters that were calculated using equation *γ* =  − *dlnω*/(3*dlnr*)

Mode	*ω*_*0*_ (cm^−1^)	*δ*_*0*_ (GPa^−1^)	δ’	$\overline{\upgamma}$
ω_2_	210	0.025	0.21	1.08
ω_6_	504	0.028	0.079	1.16
ω_3_	711	0.0069	0.056	0.29

For anisotropic 2D materials with an atomic thickness, such as MXene and graphene, two independent components of the Grüneisen tensors are usually associated with the strains that are parallel and perpendicular to the *c* axis. For simplicity, we adopted the scaling relationship proposed by Zallen et al. [38], which has been used to fit the pressure-dependent Raman shift of graphite by Hanfland et al. [28]

3 $$ \omega (P)/{\omega}_0={\left[r(P)/{r}_0\right]}^{3\gamma } $$

where *r* refers to the in-plane and out-of-plane lattice constants for intralayer and interlayer modes, respectively. *γ* is equivalent to the Grüneisen parameter that was defined in other studies [39, 40].

As only the out-of-plane modes are observed in the compression process, *c* lattice parameter as a function of hydrostatic pressure is adequate for calculation. We adopted the (002) plane’s space distance data of 0 to 26.7 GPa in Fig. 2b for the calculation of Grüneisen parameter *γ*. The averaged $\overline{\upgamma}$ up to 26.7 GPa for ω_2_, ω_6_, and ω_3_ were calculated to be 1.08, 1.16, and 0.29, respectively (Table 2). Similar to the graphite, the smaller $\overline{\upgamma}$ of ω_3_ compared with the other two modes indicates a smaller change in force constants is involved in the rigid-layer motion [28]. To the best of our knowledge, the Grüneisen parameters of Ti_3_C_2_T_*x*_ have not been reported yet. However, we can still compare our data with other 2D materials. Zha et al. reported Grüneisen parameters as 4–5 for acoustic phonon modes of Ti_2_CO_2_ [41]. Because the Grüneisen parameters of high-frequency optical modes are usually one or two orders lower than those of the low-frequency modes [28], the Grüneisen parameters for optical phonons of Ti_2_CO_2_ can be estimated to be 0.05–0.5, which are similar to those of our values for Ti_3_C_2_T_*x*_. Recently, Peng et al. [42] reported the Grüneisen parameters at room temperature as 1.22, 1.20, and 1.15 for MoS_2_, MoSe_2_, and WS_2_, respectively, which are larger than our results. Our results are also smaller than those of graphene (1.99 for *E*_*2g*_ mode) [40] and graphite (1.06 for *E*_*2g*_ mode) [28]. This finding indicates that Ti_3_C_2_T_*x*_ MXene has the weakest bonding anharmonicity among these ultrathin 2D materials [42].

## Conclusions

In conclusion, we measured lattice deformation and phonon response of Ti_3_C_2_T_*x*_ thin flakes at different hydrostatic pressures up to 26.7 GPa. No phase transformation has been observed below a pressure of 26.7 GPa. All the phonon modes show a positive frequency shift with increasing pressures. The positive Grüneisen parameters of three out-of-plane phonons are calculated to be 1.08, 1.16, and 0.29. Our results increase understanding of the mechanical and thermal properties of Ti_3_C_2_T_*x*_ at high pressures.

## Methods

Ti_3_C_2_T_*x*_ powder was prepared by a method reported by reference [43]. Briefly, Ti_3_AlC_2_ powder (Forsman, 10 g) was etched by HF solution (160 ml) at room temperature for 5 h. The obtained Ti_3_C_2_T_*x*_ powder was dispersed into DI water and ultrasonically exfoliated at a power of 700 W. The resulted solution was separated after being stored by 24 h. The obtained upper layer solution was used for further Raman, atomic force microscope (AFM), and scanning electron microscope (SEM) analyses. X-ray diffraction (XRD) spectra at ambient pressure were measured using an X-ray diffractometer (Rigaku, MiniFlex600). SEM images were obtained using a scanning electron microscope (Hitachi, Su1510). In situ high-pressure XRD measurements were performed at the Shanghai Synchrotron Radiation Facility by a gasket high-pressure diamond anvil cell (DAC) at room temperature. To produce a hydrostatic environment around the sample, we used methanol/ethanol/water (16:3:1) as a pressure transmitting medium. The pressure was determined by the pressure-dependent spectral shift of the sharp ruby fluorescence R1 line. The sample was placed in a stainless steel gasket hole (100 μm in diameter) with a diamond culet (400 μm in diameter). High-pressure Raman scattering measurements were performed using a Renishaw inVia Raman spectrometer with an excitation wavelength of 532 nm. The topographical measurements were conducted on an AFM instrument (Bruker, Innova).

The fitting of diffraction peak positions and Raman peak shifts was conducted on OriginPro package. A user-defined function, *y* = (A1 × *x* + 1)^A2^, was used by setting A1 and A2 as the fitting parameters. Proper fitting can be obtained easily by a simplex method.

## Abbreviations

2D: Two dimensional
AFM: Atomic force microscope
DAC: Diamond anvil cell
SEM: Scanning electron microscope
TMDs: Transition metal dichalcogenides
XRD: X-ray diffraction
